# Introducing the “Twilight” operating room concept: a feasibility study to improve operating room utilization

**DOI:** 10.1186/s13037-022-00335-8

**Published:** 2022-07-27

**Authors:** Bee Shan Ong, Rebecca Thomas, Simon Jenkins

**Affiliations:** 1 Department of Surgery, Lyell McEwin Hospital, Haydown Rd, Elizabeth Vale, South Australia 5112 Australia; 2 Department of Anaesthesia, Lyell McEwin Hospital, Haydown Rd, Elizabeth Vale, South Australia 5112 Australia

**Keywords:** Operating room, Surgery, Cost-effectiveness

## Abstract

**Background:**

The efficient use of operating room is important to ensure optimum cost–benefit for the hospital and to reduce elective surgery waiting times. We introduced a concept of non-commissioned “Twilight” operating room to reduce patient waiting list and mitigate consequences of non-availability of elective operative time due to closure of an affiliated hospital operating suite.

**Methods:**

A retrospective audit was performed during a 10-month period where “Twilight” operating room was implemented in our institution. Additionally, we included patients that were operated on 13 non-commissioned whole day operative sessions on Saturdays during the same period.

**Results:**

A total of 223 surgical procedures were performed during the study time window. Most patients have American Society of Anaesthesiologists (ASA) Class 2. Participating subspecialties were General Surgery, Orthopaedic surgery, Gynaecology, Urology, Plastic surgery and Dental surgery. A wide range of operations was performed in the “Twilight” operating room. No major complications were observed.

**Conclusion:**

Our study demonstrated the feasibility of conducting elective surgeries after hours with the advantage of reducing the hospital’s elective surgery waiting time. Importantly, no major post-operative complications were reported. This model is a feasible and safe strategy to restore surgical activity impacted by the COVID-19 pandemic.

## Background

The efficient use of operating room is important to ensure optimum cost–benefit for the institution and to clear elective surgery waiting lists. This is especially important in these unprecedented times of COVID-19 pandemic that has resulted in major disruption of routine hospital services globally [1]. In a global expert response study done by Nepogodiev et al.[2], an overall 12-week cancellation rates for elective surgery in 190 countries would be 72.3 percent. It was estimated that a median of 45 weeks is needed to clear the backlog of operations resulting from COVID-19 disruption if normal surgical volume is increased by 20 percent by these countries after the pandemic [2].

In general, elective surgeries are usually performed during standard office hours between 9am and 5 pm. In our institution, we have a morning session between 9am and 12.30 pm and an afternoon/PM session between 1 pm and 4.30 pm.

We introduced a new concept of ‘Twilight’ operating room, where elective cases were performed after hours between 5 pm and 8.30 pm. We hypothesise that this concept can be extrapolated to provide a feasible alternative to address global burden of cancelled surgeries due to COVID-19 pandemic.

The surgical teams in Lyell McEwin Hospital/South Australia, had adopted this concept when elective surgeries were suspended temporarily in an affiliated hospital (Modbury Hospital) for redevelopment. Participating subspecialties included general surgery, orthopaedic surgery, urology, obstetrics and gynaecology, plastic surgery and dental.

In this retrospective audit, we plan to analyse the cost-effectiveness of “Twilight” operating room as well as its impact on elective surgery waiting period. The result of this study would provide important insights into whether “Twilight” operating room is beneficial for implementation in the long term. The goal of this concept is to provide a feasible alternative to address the global burden of cancelled surgeries due to COVID-19 pandemic.

In addition, during the same period of time, we had elective operating sessions conducted on Saturdays where these sessions started at 9am and ended at 4.30 pm.

## Methods

We performed a retrospective audit using data gathered from the operating room database at our institution. We considered actual surgery start and end time and evaluated operating room efficiency by comparing them to planned surgery time. All patients operated in “Twilight” lists were included. The study was carried out between July 20, 2020 and April 8, 2021. There were no exclusion criteria in this study. There were two separate weeks in this study period that coincided with COVID-19 lockdown periods in South Australia where elective surgeries were suspended. The study protocol was assessed by Research Ethics Committee of Northern Adelaide Local Health Network and deemed a retrospective quality assurance audit project.

### Data collection

**Fig. 1:**
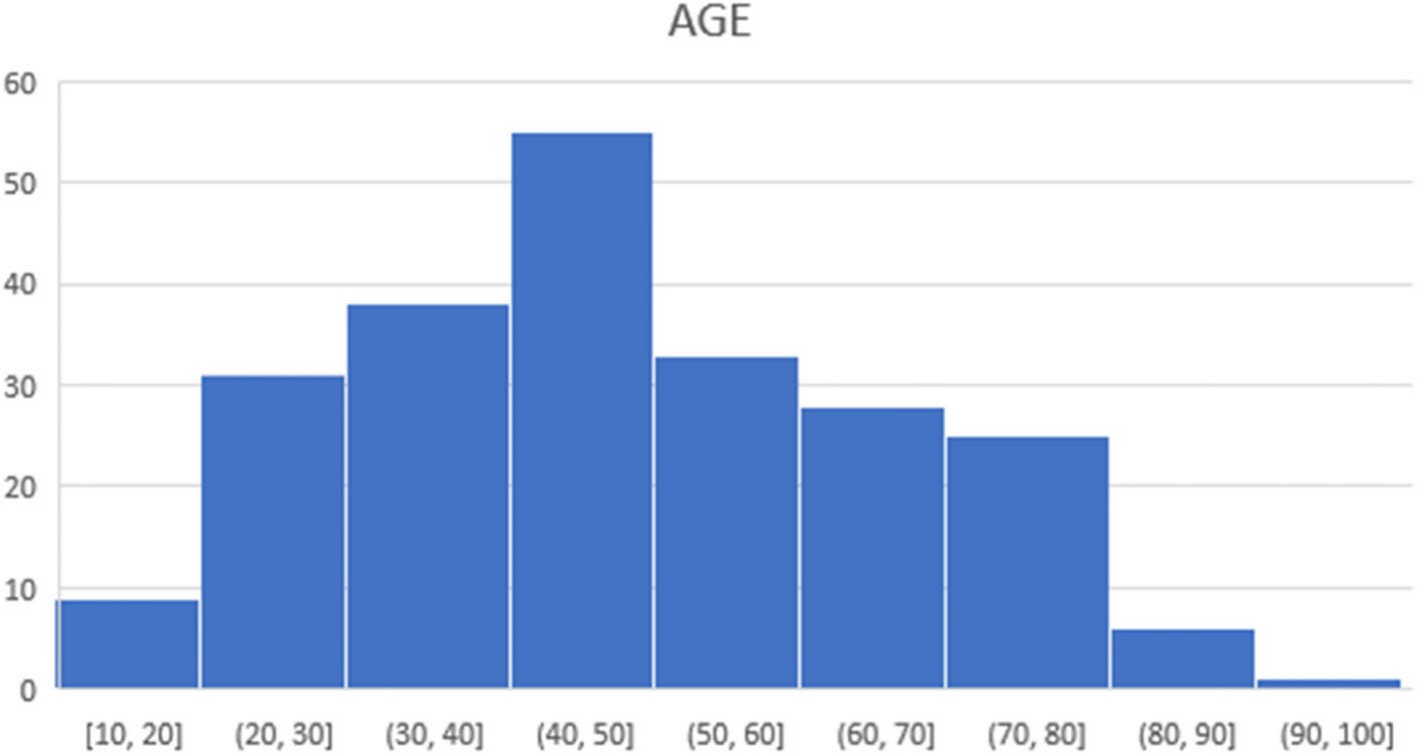
Age group of patients operated in “Twilight” sessions

**Fig. 2:**
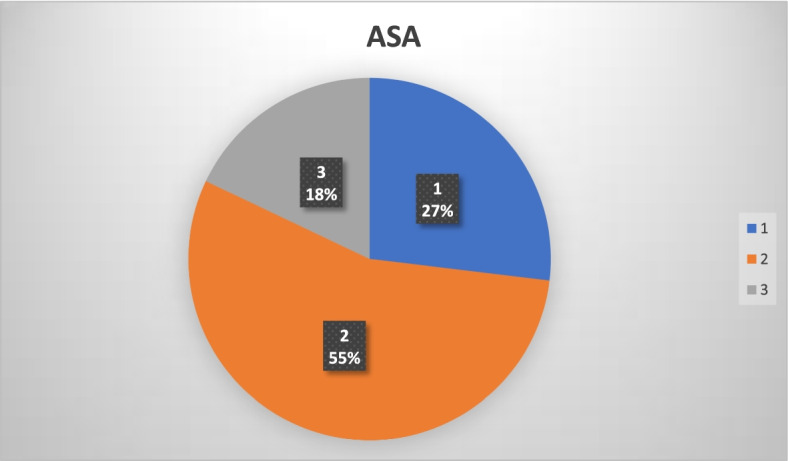
ASA classifications of patients

The following patient data were collected from electronic medical records: age, American Society of Anaesthesiology (ASA) physical status and body mass index (BMI). The following operative data were collected: type of surgery, planned surgery time as well as actual surgery start and end times. total of 223 surgical procedures were performed between July 20, 2020 and April 8, 2021. There were only three cancellations, all of which were initiated by patients. Our patients’ age ranged between 10 and 91 (Fig. 1). Though there were no selection criteria for patients to be operated in “Twilight” operating room, we observed a majority of patients with ASA 2 in our dataset (Fig. 2). In addition, our patients’ BMI ranged between 17 and 59 kg/m^2^.

**Fig. 3:**
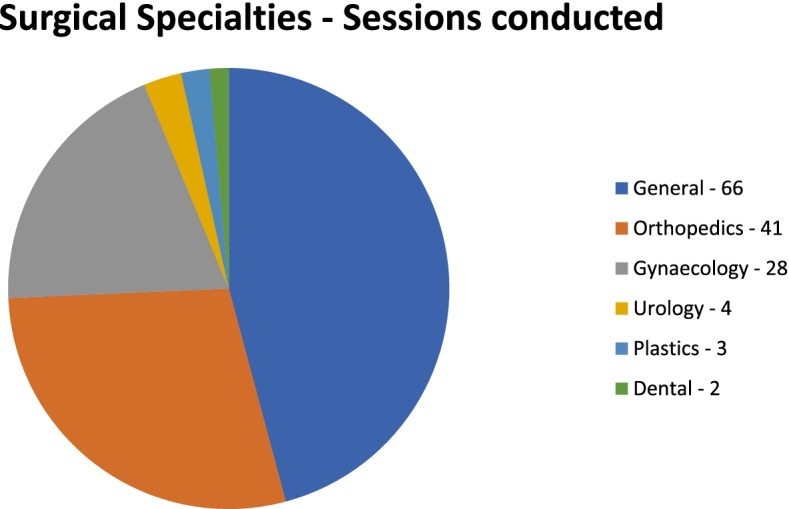
Number of sessions conducted in each surgical specialty

**Fig. 4:**
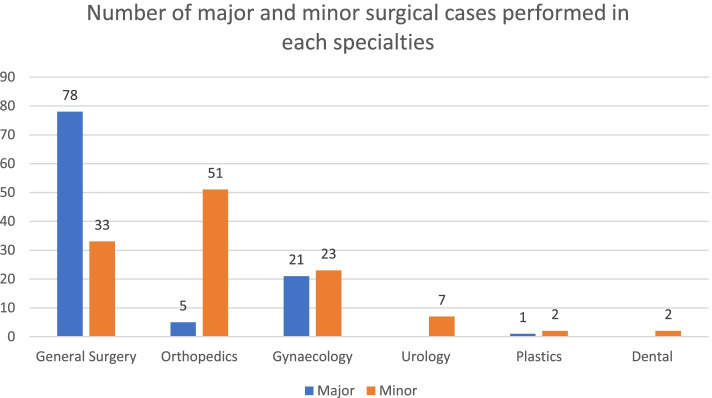
Number of major and minor surgical cases performed in each specialty

A total of 144 sessions were performed in the “Twilight” operating room. Participating subspecialties were General Surgery, Orthopaedic surgery, Gynaecology, Urology, Plastic surgery and Dental. General surgery and Orthopaedic Surgery were the two most common surgical specialties with 66 and 41 sessions performed respectively. This was followed by Gynaecology, Urology, Plastics Surgery and Dental with 28, 4, 3, and 2 sessions respectively (Fig. 3). There was no session overutilization in 102 of the sessions (70.8%). The average operating room underutilisation time was between 0 and 192 min whereas the average over utilization time was between 2 and 152 min. The common reasons for overrun were overrunning of afternoon list, delay in obtaining operating equipment and radiology staffs. Figure 4 highlighted in detail the number of major and minor surgical cases performed in each specialty. A wide range of operations was performed in the “Twilight” session (Table 1).

**Table 1:** Types of operations performed in each specialty

**General Surgery**	**Orthopaedics**
Laparoscopic Cholecystectomy	Open Reduction Internal Fixation
Umbilical / Inguinal Hernia Repairs	Anterior Cruciate Ligament Reconstruction
Excision of Pilonidal Sinus	Arthroscopies
Laparotomy and Small Bowel Resection	Rotator Cuff Repair
Endoscopy, Colonoscopy	Meniscal Repair/ Debridement
Hemorrhoid Artery Ligation/ Hemorrhoidectomy	Carpal Tunnel Release
Laparoscopic Right Hemicolectomy	**Gynaecology**
Examination Under Anaesthesia	Cystectomy
Closure of Ileostomy	Hysteroscopy + Dilatation and Curettage
Carpal Tunnel Release	Excision of Endometriosis
**Urology**	Mirena Insertion
Orchidectomy	Hysterectomy
Cystoscopy / Pyeloscopy/ Ureteroscopy	Salpingectomy
Transurethral Resection of Bladder Tumour/ Prostate	Oophorectomy
Ureteric Stent Placement	Myomectomy
**Plastic Surgery**	Anterior / Posterior Vaginal Repair
Excision of Squamous Cell Carcinoma of Ear with FLAP	**Dental**
Excision of Recurrent Infra-auricular Basal Cell Carcinoma, Superficial Lower Parotidectomy and Cervio-Facial FLAP	Left Revision Mastoidectomy
Division of left lower lid Chondromucosal FLAP with Insetting	Tonsillectomy

More importantly, no complications from surgeries were observed in our study.

As for the Saturdays, there were a total of 13 sessions performed with 5 of them being General Surgery list, 4 being Urology, another 4 sessions for Gynaecology. Out of these 13 sessions, 3 sessions overrun with the average overutilization time between 10 and 75 min.

## Discussion

The traditional operating rooms in Australia work between 9 am and 4.30 pm. In our institution with 9 operating rooms, traditionally 7 of them remain unused from 5 pm till 9am the next day except for one operating room used for emergency general surgical cases and a second operating room for emergency obstetric cases.

However, with modernisation of a subsidiary hospital (Modbury Hospital) requiring closure of the operating rooms, it was necessary to find innovative ways to boost productivity and reduce elective surgery waiting list. This period also coincided with the COVID-19 pandemic with two lockdown periods in South Australia (March 22, 2021; November 19,2020).

Since the traditional day elective surgery was no longer relevant for the current circumstances in our institution, in order to improve productivity and reduce the elective surgery wait list, it was decided to trial “Twilight” session between 5 pm and 8.30 pm on three weekdays a week and a Saturday operative session running between 8.30 am and 5 pm.

There is no literature reporting on cost-effectiveness or financial burden of after-hours elective surgery. Our study is also unique as it included many surgical subspecialties.

Most published studies made comparison between surgeries performed during hours and after-hours or between surgeries performed during weekdays or weekends [3, 4]. Majority of them had post-operative mortality as their primary outcome [4, 5].

Operating room services represent a significant proportion of hospital costs. In 2011–12, approximately 210,000 patients in New South Wales had elective surgery accounting for 45% of all public admissions [6]. This is estimated to cost approximately $1.3 billion each year or about 17% of New South Wales Health’s inpatient services budget [6].

The efficient use of operating room is important to ensure optimum cost–benefit for the hospital and to clear waiting lists. The NHS Management Executive recommends that hospitals should aim to use 90% of planned operating room time and its utilisation should be used as a key performance indicator [7].

In a 2010 study done by Florence et al. over a 5 year-period at a single academic medical centre, patients undergoing non-emergent general and vascular surgeries at night (between 7 pm and 6.45am) were not found to have an increased postoperative morbidity or mortality risk. It was suggested that performing non-emergent surgeries at night is a safe solution for daytime overcrowding of operating theatre [8].

On the contrary, a recent systematic review performed by Cortegiani et al. identified that out-of-hours surgery was associated with a higher post-operative risk of death than daytime surgery. This study, however, combined both emergency and elective surgeries and hence may not truly reflect the risk associated with after-hours elective surgery alone [9].

As stated by Divatia et al., personal accountability, streamlining of procedures, interdisciplinary teamwork, and accurate data collection including regular audit are all important contributors to improve operation room efficiency [10].

Extensive research has been done on operating room utilization and the factors affecting its efficiency but data on ways to improve operating room utilization is limited. To our best knowledge, the concept of “Twilight” operating room is unique and hasn’t been implemented elsewhere in Australia. It is anticipated that this new concept has comparable outcome to day surgery in addition to the added benefit of reducing waiting period with minimal impact on cost incurred for extra staffs and overtime.

Other factors to consider are radiology and laboratory availability for certain surgical procedures that require image intensifier and frozen sections to be carried out.

Our study demonstrated the feasibility of conducting elective surgeries after hours with the advantage of clearing hospital waiting list. The waiting list in our institution was completely cleared for July 2020 to April 2021 with no overdue elective surgeries. Importantly, no post-operative complications were reported. This model is a feasible and safe strategy to restore surgical activity impacted by COVID-19 pandemic.

The limitations of this model include conversion of day surgery cases to overnight stay due to the late operative hours which increases bed occupancy and nursing cost. Moreover, the running of “Twilight” sessions was dependent on completion of pm sessions. This is an important limitation to acknowledge as surgical start time is widely acknowledged as a crucial target for efficiency savings [11]. In addition, not all body mass index was collected for our patients.

We have a few suggestions to address the shortcomings of our “Twilight” sessions. Firstly, a dedicated operating room with a group of hospital staffs for "Twilight” sessions will ensure that the start time of “Twilight” session is independent of previous pm session. Alternatively, combining pm and "Twilight” session under a surgeon so that the surgeon can adjust the list accordingly can be considered. In addition, having radiology staffs in operating room will reduce logistic delay.

A normal 3.5 h commissioned session on Monday to Friday during normal hours costs the hospital AUD 4309.51 while these un-commissioned additional “Twilight” lists or Saturday 3.5 h sessions incurred AUD 5841.44. This can be considered cost effective in view of elimination of the overdue patient waiting list, patient anxiety and distress and progression of disease.

## Conclusion

We have highlighted the feasibility and cost effectiveness of performing elective surgeries after hours. The experience of implementing these innovative measures can be utilised in the future in the events of any catastrophe or epidemic / pandemics resulting in similar extenuating situations.

## Data Availability

Not applicable.
